# Curcumin and doxorubicin encapsulated in biocompatible clay‐based nanomaterial: A strategy to overcome multidrug resistance

**DOI:** 10.1002/ardp.202400702

**Published:** 2024-12-26

**Authors:** Paola Poma, Marina Massaro, Salvatrice Rigogliuso, Lucia Condorelli, Rita Sánchez‐Espejo, César Viseras, Monica Notarbartolo, Serena Riela

**Affiliations:** ^1^ Dipartimento di Scienze e Tecnologie Biologiche, Chimiche e Farmaceutiche (STEBICEF) Università di Palermo Palermo Italy; ^2^ Department of Pharmacy and Pharmaceutical Technology, Faculty of Pharmacy University of Granada Granada Spain; ^3^ Andalusian Institute of Earth Sciences, CSIC‐UGR Armilla Granada Spain; ^4^ Dipartimento di Scienze Chimiche (DSC) Università di Catania Catania Italy

**Keywords:** 3D model, clay mineral, curcumin, nanomaterials, P‐glycoprotein inhibition

## Abstract

Multidrug resistance (MDR) due to the overexpression of the P‐glycoprotein (P‐gp) efflux pump remains a significant challenge in cancer therapy, also in breast cancer. Traditional pharmacological approaches have focused on using inhibitors to modulate P‐gp expression and function. Curcumin, a polyphenol derived from *Curcuma longa* L., is one of the most extensively studied natural compounds with the potential as an effective P‐gp inhibitor. Despite its promising attributes, the clinical application of P‐gp inhibitors is complicated by P‐gp's presence in healthy cells, such as those in the intestinal barrier and blood–brain barrier, which can lead to increased toxicity. To address these challenges, we developed a novel multifunctional nanomaterial by covalently bonding halloysite nanotubes (HNTs) with hectorite (Ht) and loading it with curcumin and doxorubicin. The efficacy of the co‐delivery of curcumin and doxorubicin by HNTs‐Ht nanomaterial was evaluated by cytotoxicity assays on MCF‐7R cells, both in two‐dimensional (2D) and in three‐dimensional (3D) models. The obtained data show that curcumin causes increased doxorubicin accumulation by acting as a substrate for P‐gp transport and as a stimulator of the adenosine triphosphate (ATP)‐dependent drug efflux transporter on a doxorubicin‐resistant breast cancer cell line. The results suggest that the HNTs‐Ht nanomaterial could provide a promising approach to improve chemotherapy effectiveness by overcoming MDR and enhancing treatment outcomes.

## Graphical abstract

The combination of curcumin and doxorubicin in a multifunctional delivery system represents a promising strategy to address multidrug resistance in breast cancer. Curcumin causes increased doxorubicin accumulation by acting as a substrate for P‐gp transport and as a stimulator of the ATP‐dependent drug efflux transporter.

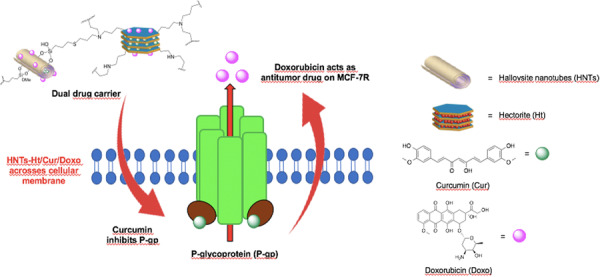

## 1 INTRODUCTION

To date, the pharmacological approach to overcome multidrug resistance (MDR) caused by the overexpression of the P‐glycoprotein (P‐gp) efflux pump has primarily focused on using inhibitors to reduce its expression and/or its function. There is now a substantial list of potential inhibitors, particularly fourth‐generation ones that are natural molecules with pleiotropic actions. These often modulate both the expression and activity of the efflux pump^[^ ^1^ ^]^ which is crucial in managing MDR in various tumor types, including breast cancer.^[^ ^2^, ^3^, ^4^, ^5^ ^]^

Curcumin, a polyphenol extracted from the dried rhizomes of *Curcuma longa* L., stands out as one of the most studied natural molecules. Its multitarget mechanism of action makes it a potential chemotherapeutic agent and an effective P‐gp inhibitor.^[^ ^6^, ^7^ ^]^ The significance of using natural compounds like curcumin lies in their lower toxicity and ability to target multiple pathways simultaneously, making them ideal candidates for overcoming MDR. Curcumin and its analogs and derivatives have been extensively investigated for their effects on breast cancer.^[^ ^8^, ^9^, ^10^ ^]^

However, a significant obstacle to the clinical application of P‐gp inhibitors is that P‐gp is naturally present in healthy cells, such as those in the intestinal barrier and blood–brain barrier (BBB). Inhibiting the physiological function of P‐gp can lead to increased toxicity due to higher drug absorption and reduced excretion of metabolites. To address this, researchers have explored the benefits of multifunctional delivery systems that combine the pump inhibitor with substrate drugs, such as doxorubicin.^[^ ^11^ ^]^ These systems often utilize nanoparticles or nanosuspensions, but among the emerging strategies, clay mineral‐based nanomaterial have gained attention due to their unique properties.^[^ ^12^, ^13^, ^14^, ^15^, ^16^ ^]^

Halloysite, an aluminosilicate with a hollow tubular structure (HNTs), is particularly effective in binding molecules both on its external surface and in its internal cavity or lumen.^[^ ^17^, ^18^, ^19^ ^]^ This unique structure offers several significant advantages in drug delivery systems. First, it allows for the efficient loading and controlled release of therapeutic agents, enhancing their efficacy.^[^ ^15^, ^20^ ^]^ The high aspect ratio, large surface area of HNTs further contribute to improving the stability and effectiveness of the delivered drugs. Additionally, the excellent mechanical strength of halloysite adds robustness and durability to the drug delivery system. Another key advantage is its ability to disperse in aqueous media, increasing its versatility and applicability in various biomedical application.^[^ ^21^ ^]^ Its biocompatibility ensures that it is safe for use in the human body,^[^ ^22^ ^]^ reducing the risk of adverse reactions. Furthermore, halloysite exhibits high cellular uptake, with a propensity to localize in the perinuclear region of cells.^[^ ^23^ ^]^ This localization is particularly advantageous for drug delivery, as it brings therapeutic agents closer to the cell nucleus, where they can exert their effects more effectively.

Hectorite (Ht), another natural clay mineral from the smectite group, possesses a lamellar structure that contributes to its high surface area and excellent cation exchange capacity. This lamellar configuration allows for the intercalation of drugs and other bioactive molecules, facilitating their sustained release and enhancing their bioavailability.^[^ ^24^ ^]^ Ht high swelling capacity in water and its ability to form stable colloidal dispersions are crucial for drug delivery applications, as they ensure a uniform distribution of the therapeutic agents.^[^ ^25^, ^26^ ^]^

Combining the properties of HNTs and Ht presents a promising strategy for developing advanced drug delivery systems. The synergistic integration of these nanomaterials can significantly enhance the stability, efficacy, and targeted delivery of therapeutic agents, making it an effective approach for overcoming MDR and improving treatment outcomes.

In our study, we explore the mechanisms of action of curcumin on the P‐gp function in an MDR breast cancer cell line, MCF‐7R. We synthesized a novel multifunctional nanomaterial based on halloysite covalently bonded to hectorite (HNTs‐Ht) and loaded it with curcumin and doxorubicin. We then tested the HNTs‐Ht system in an MDR cell line for the first time, aiming to evaluate its potential effectiveness in overcoming drug resistance and improving therapeutic efficacy.

This nanomaterial showed a triple advantage: evading the efflux pump via cell uptake through endocytosis rather than passive diffusion, synergistically increasing the accumulation and toxicity of the chemotherapeutic drug, and potentially targeting the tumor cells.

We also compared the cytotoxic effects of curcumin in combination with doxorubicin to those of the delivery system, in two‐dimensional (2D) and three‐dimensional (3D) multidrug‐resistant breast cancer cells (MCF‐7R).

## 2 RESULTS AND DISCUSSION

In this section, we present and discuss the findings of our study, which explores the efficacy of a novel nanocarrier system based on clay minerals for the delivery of curcumin and doxorubicin in overcoming MDR in breast cancer cells. Our investigation aims to elucidate the synergistic effects of curcumin and doxorubicin encapsulated in this biocompatible nanomaterial, focusing on their potential to enhance therapeutic outcomes by targeting the P‐gp efflux pump and modulating critical cellular pathways involved in drug resistance. First of all, since up to now, to the best of our knowledge, the effects of curcumin on P‐gp function are not clear, we performed biological assays to validate them. Afterward, to overcome limitations associated with the potential clinical use of curcumin, a carrier system based on Ht covalently linked to halloysite was synthesized and characterized, and then it was loaded with curcumin and doxorubicin to obtain the final nanomaterial HNTs‐Ht/Cur/Doxo. Finally, we evaluated the antiproliferative activity of the HNTs‐Ht/Cur/Doxo nanomaterial through a series of in vitro experiments on 2D and 3D MDR breast cancer models.

### 2.1 Effects of curcumin on P‐gp function

**Figure 1:**
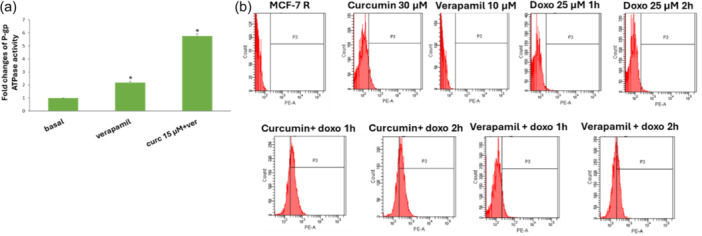
(a) Effects of curcumin on verapamil‐stimulated P‐gp ATPase activity. The data are expressed as fold changes of P‐gp ATPase activity compared with basal one (ΔRLU_TC_/ΔRLU_basal_) and are presented as mean ± SE of two experiments, each in duplicate. *Differences when treatments are compared to the basal activity *p* < 0.01 (one‐way ANOVA followed by Tukey's test). (b) Effects of curcumin on intracellular accumulation of doxorubicin in MCF‐7R cell line. Representative example of flow cytometry analysis. The cells were treated with curcumin 30 µM or verapamil 10 µM. After 24 h of incubation, doxorubicin 25 µM (13.6 µg/mL) has been added for different times (1 and 2 h). ANOVA, analysis of variance; ATPase, adenosine triphosphatase; P‐gp, P‐glycoprotein.

One of the many qualities of curcumin is that this molecule is able to modulate the function of the pump; in fact, it increased verapamil‐stimulated P‐gp ATPase activity, probably acting as substrates for transport by P‐gp and stimulators of ATP‐dependent drug efflux transporter (Figure 1a). Verapamil, in fact, is a P‐gp substrate that stimulates P‐gp ATPase activity and serves as a positive control for drug stimulation of P‐gp ATPase activity. Based on these results, we performed the accumulation assay of P‐gp substrate doxorubicin by flow cytometry analysis (Figure 1b and Table 1).

**Table 1:** The intracellular accumulation of doxorubicin was measured by flow cytometric analysis.

Treatments	Fluorescence (%)
MCF‐7R (control)	0.0 ± 0.0
Curcumin 30 µM	7.3 ± 1.0^a,^ *
Verapamil 10 µM	0.0 ± 0.0^a^
Doxorubicin 25 µM 1 h	1.7 ± 0.4^a^
Doxorubicin 25 µM 2 h	2.8 ± 0.6^a^
Curcumin 30 µM + doxorubicin 25 µM 1 h	40.1 ± 11.6^b,^ **
Curcumin 30 µM + doxorubicin 25 µM 2 h	37.3 ± 9.3^b,^ **
Verapamil 10 µM + doxorubicin 25 µM 1 h	8.5 ± 3.0^a,^ *
Verapamil 10 µM + doxorubicin 25 µM 2 h	32.0 ± 9.0^b,^ **

*Note*: Different letters (a and b, *p* < 0.001) represent significant differences among the treatments (one‐way ANOVA followed by Tukey's test).

* *p* < 0.005

** *p* < 0.001 versus the control.

To do this, subcytotoxic concentrations of curcumin, specifically 15 µM for the P‐gp ATPase activity (Figure 1a) and 30 µM for the intracellular accumulation assay (Figure 1b and Table 1) were chosen. Cells were incubated for 24 or 48 h, to ensure the blocking of the pump, closely related to the resistant cell model used, followed by incubation with doxorubicin, for shorter times.^[^ ^27^, ^28^, ^29^ ^]^

The results indicate an increase in fluorescence in MCF‐7R cells after pretreatment with curcumin, which proves an intracellular accumulation of doxorubicin.

In the sensitive cell line, as expected, pretreatment with curcumin does not cause accumulation of doxorubicin compared with its control, confirming a selective mechanism of curcumin on P‐gp which is overexpressed only in the MDR cell line (Supporting Information S1: Figure S1).

Although there is an increase in doxorubicin uptake induced by curcumin, this does not determine a synergistic antitumor effect, recording only a good additivity with their combination in terms of antiproliferative action.

### 2.2 Synthesis and characterization of the HNTs‐Ht nanomaterial

**Scheme 1:**
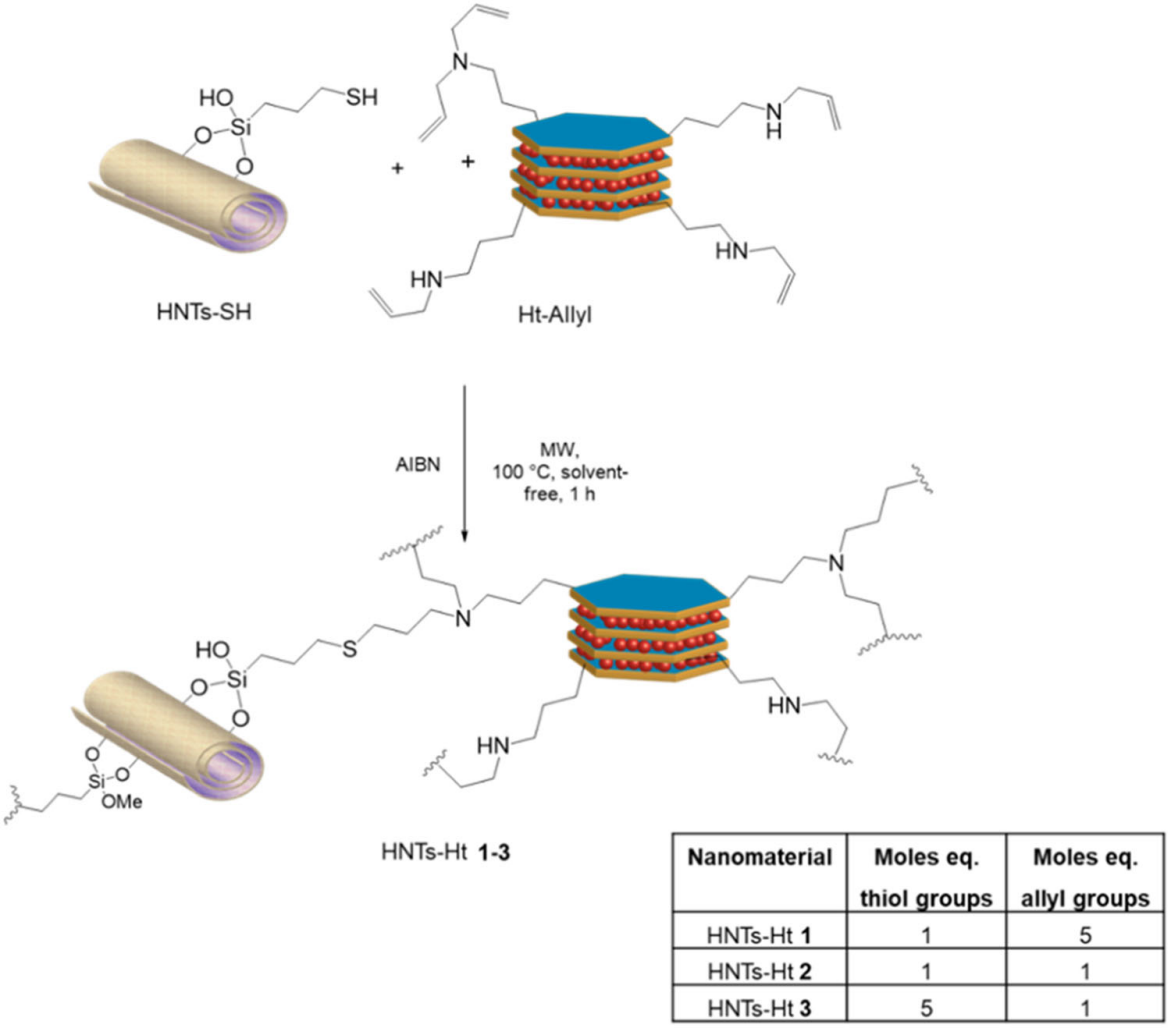
Schematic representation of the synthesis of HNTs‐Ht **1–3** nanomaterials. HNTs, halloysite nanotubes; Ht, hectorite.

The covalently linked HNTs‐Ht nanomaterial was prepared by reacting the Ht‐Allyl nanomaterial (degree of functionalization of 2.08 mmol g^–1^) with HNTs‐SH (degree of functionalization of 0.3 mmol g^–1^) by AIBN‐catalyzed thiol‐ene reaction under MW irradiation at temperature of 100°C in solvent‐free conditions for an irradiation time of 1 h, following a procedure reported elsewhere.^[^ ^30^ ^]^ For this synthesis, the reactants were mixed in different proportions as reported in Scheme 1 affording the HNTs‐Ht **1**, HNTs‐Ht **2**, and HNTs‐Ht **3** nanomaterials, respectively.

According to the mole ratio, of thiol and allyl groups on HNTs‐SH and Ht‐Allyl nanomaterials, used, the three nanomaterials could possess different morphologies and different properties. To assess this hypothesis, the obtained nanomaterials were characterized by several techniques. In particular, the aqueous mobility of the nanomaterials was investigated by dynamic light scattering (DLS) measurements. This parameter is of fundamental importance for the biological application of nanomaterials. DLS measurements allow the determination of the structural characteristics of the nanomaterials by monitoring their mobility in water and by measuring the average translational diffusion coefficient. This coefficient considers the dimension, shape, and hydration of the diffusing particles and also the existence of aggregation phenomena. By applying the Stokes–Einstein equation it is possible to calculate the average diameter (*D*_h_) of the equivalent sphere, which can be considered as an index to follow the changes in particle dimensions and interparticle aggregation. The obtained results are reported in Table 2.

**Table 2:** Average size, and ζ‐potential values for HNTs‐Ht 1, HNTs‐Ht 2, and HNTs‐Ht 3 nanomaterials.

	*D*_h_/nm	ζ−potential/mV
HNTs	295	–18
Ht	432	–36.9
HNTs‐Ht **1**	453	–17
HNTs‐Ht **2**	353	–20
HNTs‐Ht **3**	378	–20.4

Abbreviations: HNTs, halloysite nanotubes; Ht, hectorite.

**Figure 2:**
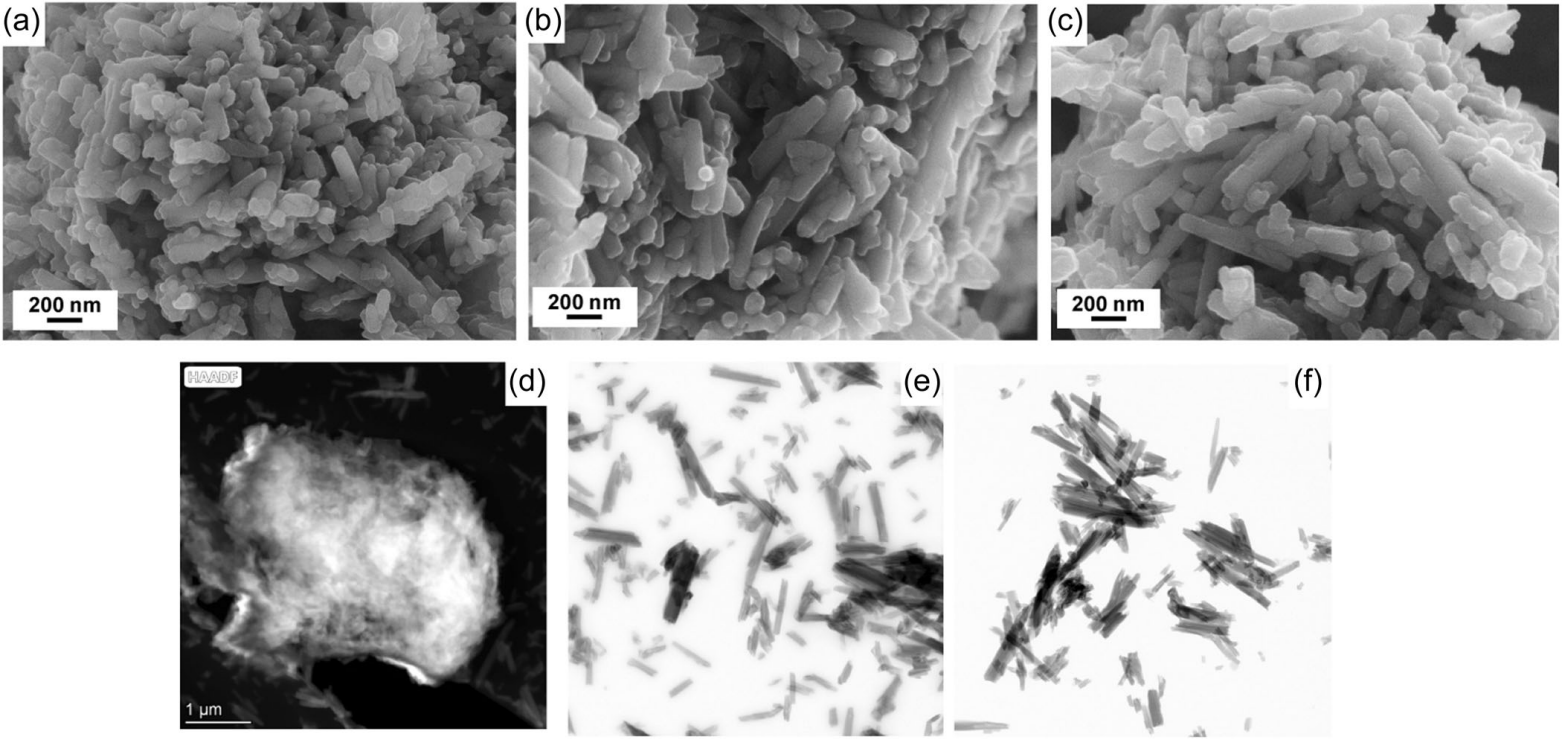
(a–c) SEM and (d–f) TEM images of (a–d) HNTs‐Ht **1**; (b–e) HNTs‐Ht **2**, and (c–f) HNTs‐Ht **3** nanomaterials. HNTs, halloysite nanotubes; Ht, hectorite.

As it is possible to note, the HNTs‐Ht **1** nanomaterial presents the highest *D*_h_ whereas the HNTs‐Ht **2** and HNTs‐Ht **3** ones show similar values. As already reported,^[^ ^30^ ^]^ taking into account the mole ratio between –SH and allyl groups in HNTs‐SH and Ht‐Allyl for the synthesis of HNTs‐Ht **1** nanomaterial, allyl groups on Ht were in excess relative to the moles of –SH groups onto HNTs therefore the double bonds that do not react in the thiol–ene reaction can undergo a self‐addition reaction, leading to the formation of a coating onto HNTs surface, constituted by a Ht cross‐linked network. This translates in slightly higher *D*_h_ value compared with pristine components. On the contrary, in the case of HNTs‐Ht **2** and HNTs‐Ht **3** nanomaterials, where the thiol groups are stoichiometric to the allyl ones or in slight excess, a nanomaterial with a less compact structure, thus with higher diffusion in aqueous media, is obtained. These findings were also confirmed by ζ−potential measurements. HNTs‐Ht **2** and HNTs‐Ht **3** nanomaterials present a more negative ζ−potential value in comparison to HNTs‐Ht **1**, indicating a better stability in aqueous medium, as a consequence of smaller dimension than HNTs‐Ht **1**, in agreement with DLS results. The morphology of the synthesized nanomaterial was imaged by scanning electron and transmission electron microscopies (SEM and TEM, respectively) (Figure 2). SEM investigations showed that all nanomaterials present a compact structure where nanotubes are clearly observable. Conversely, TEM investigations allow to detect the different morphology of the nanomaterials in agreement with DLS and ζ−potential measurements. As reported in the literature, HNTs‐Ht **1** shows a rather compact structure with a dimension of ca. 135 nm where the tubes are covered by Ht^[^ ^30^ ^]^ (Figure 2d), conversely both HNTs‐Ht **2** and HNTs‐Ht **3** nanomaterials showed a morphology mainly tubular, in agreement with the two clay dimensions (ca. 1 μm in length for HNTs and 45 nm in diameter for Ht).^[^ ^25^ ^]^

Bearing in mind the final biological application of the obtained nanomaterials, since an ideal carrier should not possess any cytotoxicity, we performed some preliminary antiproliferative assays on two different cancer cell lines, namely HL‐60 and MCF‐7. As it is possible to note from Table 3, HNTs‐Ht **1** nanomaterial showed cytotoxicity with an IC_50_ value of 0.09 and 0.7 mg mL^–1^, in HL‐60 and MCF‐7 cells lines, respectively, whereas both HNTs‐Ht **2** and HNTs‐Ht **3** resulted to be not toxic. It was hypothesized that the different results obtained could be due to the different size of the nanomaterials.

**Table 3:** IC_50_ values of the HNTs‐Ht‐based nanomaterials.

Nanomaterials	IC_50_ HL‐60 (mg mL^–1^)	IC_50_ MCF‐7 (mg mL^–1^)
HNTs‐Ht **1**	0.090 ± 0.007	0.7 ± 0
HNTs‐Ht **2**	/	/
HNTs‐Ht **3**	/	/

Abbreviations: HNTs, halloysite nanotubes; Ht, hectorite.

### 2.3 Loading of Cur and Doxo onto HNTs‐Ht nanomaterial

**Scheme 2:**
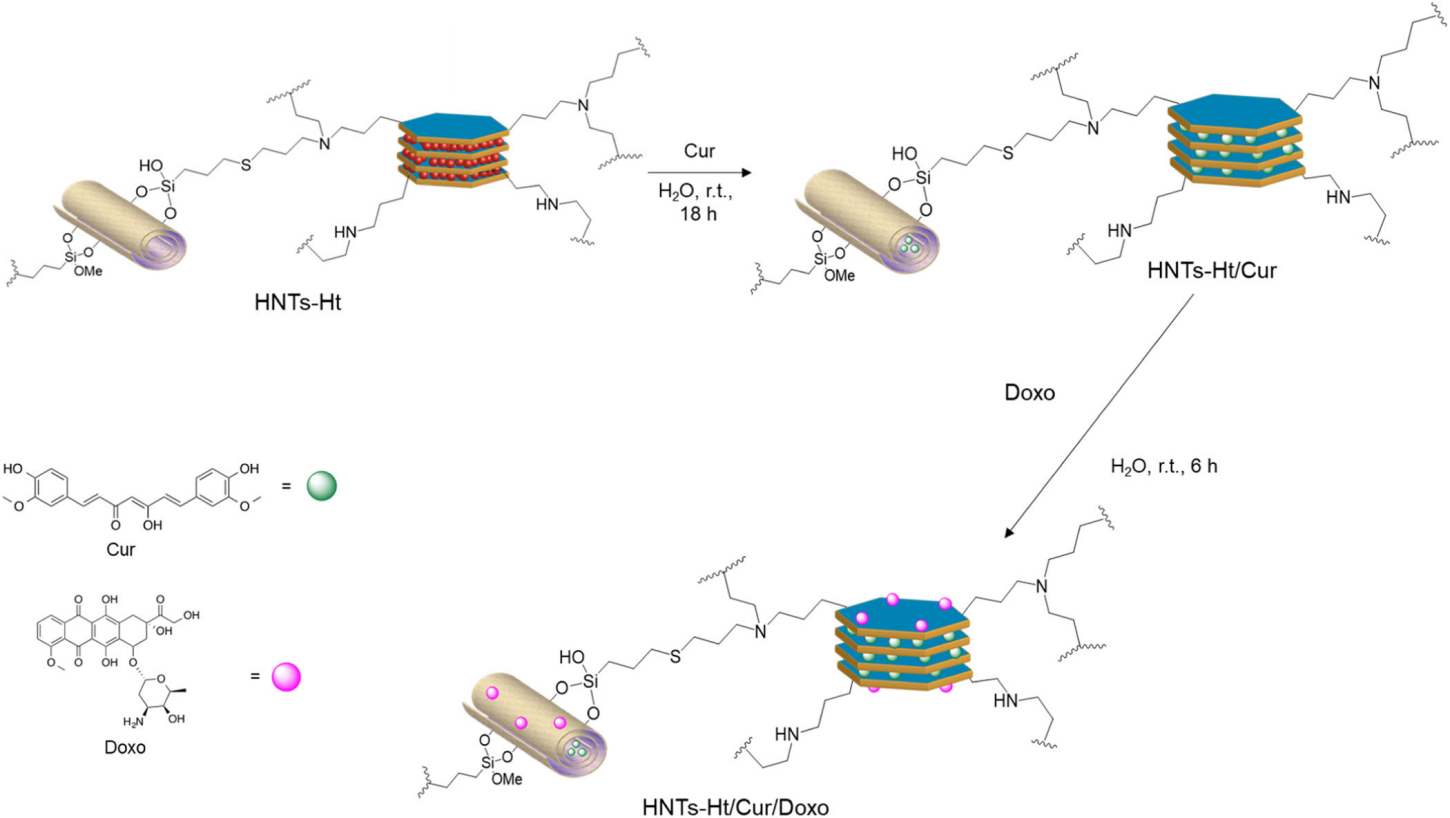
Schematic representation of the synthesis of HNTs‐Ht/Cur/Doxo nanomaterial. HNTs, halloysite nanotubes; Ht, hectorite.

In light of these findings, HNTs‐Ht **2**, hereinafter named HNTs‐Ht, was chosen as a carrier for the simultaneous delivery of both curcumin and doxorubicin. As reported in the literature curcumin interacts with HNTs lumen while doxorubicin is loaded on the negatively charged halloysite external surface.^[^ ^31^ ^]^ Preliminary investigations showed that Ht is capable to load ca. 3 wt% of curcumin as estimated by both UV–*Vis* spectroscopy and thermogravimetric analysis (TGA) (see Supporting Information), whereas doxorubicin will presumably be loaded on the negatively charged basal T‐face. Thus, the HNTs‐Ht/Cur/Doxo nanomaterial was obtained by firstly loading curcumin and then the doxorubicin (Scheme 2). Briefly, using a common strategy for loading active species inside HNTs carriers,^[^ ^15^ ^]^ the loading of the curcumin onto the nanomaterial was carried out. In detail, a dispersion of HNTs‐Ht nanomaterial (5 mL, water) was mixed with a concentrated curcumin solution (MeOH; 10^−2^ M, 2 mL). Afterward, the dispersion was evacuated for 3−5 min to promote the loading of the molecule into the HNTs lumen and left to stir at room temperature for 18 h. After work‐up, the nanomaterial (HNTs‐Ht/Cur) showed a drug loading of ca. 7.3 wt% with an entrapment efficiency of ca. 99%, as highlighted by UV–*Vis* measurements. The obtained HNTs‐Ht/Cur was used as a scaffold for the loading of doxorubicin obtaining the final nanomaterial HNTs‐Ht/Cur/Doxo which showed a doxorubicin loading of ca. 0.4 wt%. The successful loading was also verified by FT‐IR spectroscopy, which showed, beside the typical vibration stretching bands of the two clay minerals, some new bands related to the presence of the drugs in the final nanomaterial (Supporting Information S1: Figure S3).

### 2.4 Evaluation of the antiproliferative effects of HNTs‐Ht/Cur/Doxo in 2D MCF‐7R

**Figure 3:**
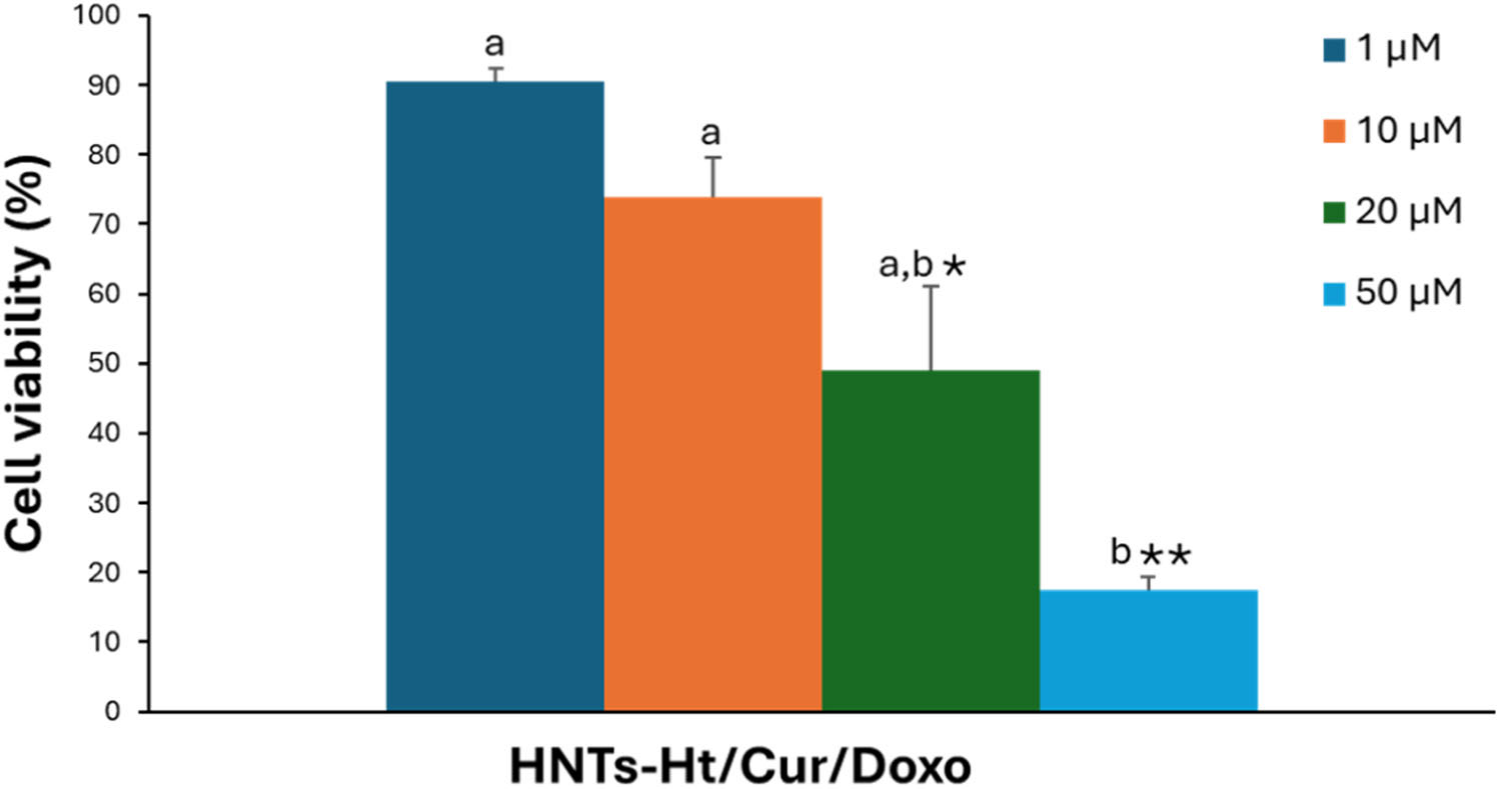
Cytotoxic activity of HNTs‐Ht/Cur/Doxo nanomaterial. Cells were exposed to nanomaterial for 72 h, at different concentrations and viability was assessed by cell counting. Data are expressed as mean ± standard error of two experiments performed in duplicate. *p < 0.05 and **p < 0.005 versus the control. Different letters (a and b) represent significant differences in cytotoxic activity among the concentrations (*p* < 0.05, one‐way ANOVA followed by Tukey's test). ANOVA, analysis of variance; HNTs, halloysite nanotubes; Ht, hectorite.

To analyze the antiproliferative power of the nanomaterial loaded with curcumin and doxorubicin, we conducted a Trypan blue exclusion test of cell viability, after 72 h of treatment. Figure 3 shows the trend of the percentage of cytotoxicity at four different concentrations (IC_50_ = 20.0 ± 2.8 µM). This IC_50_ equates exactly to the concentration of curcumin present in the vehicle which is congruent with the concentration of doxorubicin of 0.4 µg/mL. Both concentrations alone are subcytotoxic (IC_50_ values of 30 and 68 µM for curcumin and doxorubicin, respectively).

**Figure 4:**
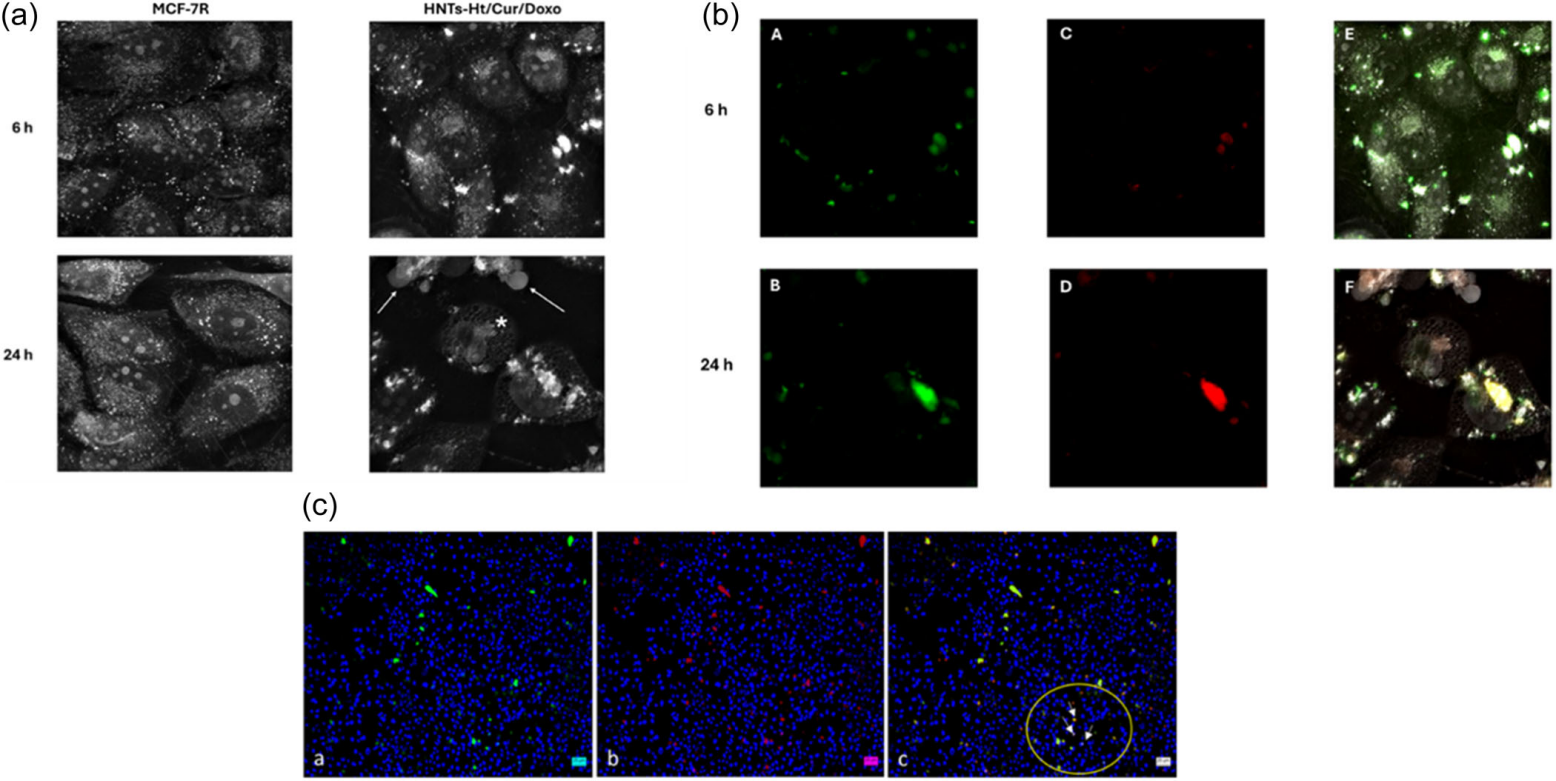
(a) Nanolive image of MCF‐7R cells (control and treated with nanomaterial at 6 and 24 h). The presence of apoptotic bodies is indicated by the arrows, while the asterisk indicates the vacuoles present in the cytoplasm. (b) Representative images of compounds localization in MCF‐7R cells after 6 and 24 h of treatment with HNTs‐Ht/Cur/Doxo nanomaterial. Curcumin (a, b) doxorubicin (c, d) and merged (e, f) fluorescence microscopy images. (c) Immunofluorescence of MCF‐7R cells after treatment with nanomaterial for 24 h (a, b, c). The nuclear co‐localization of two compounds, curcumin and doxorubicin, is clearly shown by white arrows in panel c. HNTs, halloysite nanotubes; Ht, hectorite.

The images obtained with microscopy analysis point out the uptake of the nanomaterial HNTs‐Ht/Cur/Doxo nanomaterial inside the MCF‐7R cells (Figure 4). After 6 h of exposure to HNTs‐Ht/Cur/Doxo, the cells are clearly in a stressed status, which increases after 24 h posttreatment, in fact, numerous stress vacuoles are visible and the apoptotic bodies around the cells confirm the synergistic antitumor action of the two molecules (Figure 4a). In Figure 4(b,c) is possible to observe the co‐localization of the two molecules; after 6 h curcumin and doxorubicin have cytoplasmatic localization, which becomes perinuclear after 24 h.

### 2.5 Evaluation of the antiproliferative effects of HNTs‐Ht/Cur/Doxo in 3D MCF‐7R

**Figure 5:**
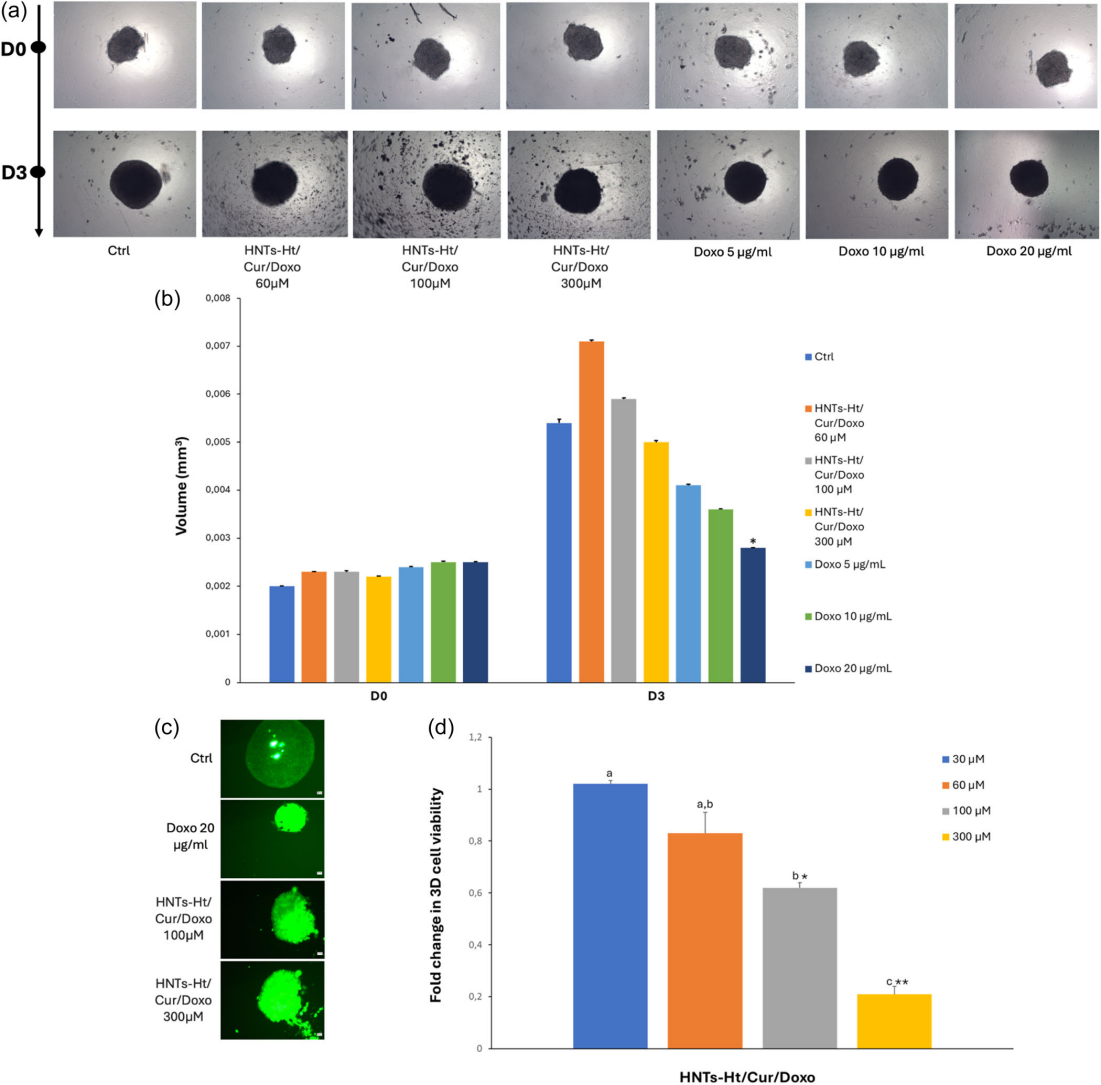
(a) Representative images of three‐dimensional tumor spheroid cultures of MCF‐7R (optical microscope AxioVert200 Zeiss, 5× magnification). (b) Volumes of three‐dimensional (3D) tumor spheroid are expressed as mean ± standard error of two experiments performed in duplicate. *p < 0.05 versus the control D3 (one‐way ANOVA followed by Tukey's test). (c) Representative images of 3D tumor spheroid cultures of MCF‐7R after incorporation of RealTime‐Glo MT Cell Viability Assay dye (Optical microscope AxioVert200 Zeiss, 5× magnification). (d) Cytotoxic activity of HNTs‐Ht/Cur/Doxo evaluated in 3D MCF‐7R. Cells were exposed to nanomaterial for 72 h, at different concentrations and viability was assessed by RealTime‐Glo MT Cell Viability Assay. The data are expressed as fold changes in cell viability compared to control and are presented as mean ± SE of two experiments. *p < 0.05 and **p < 0.005 versus the control; different letters (a, b and c) represent significant differences in cell viability among the concentrations (*p* < 0.05, one‐way ANOVA followed by Tukey's test). ANOVA, analysis of variance; HNTs, halloysite nanotubes; Ht, hectorite.

Three‐dimensional tumor spheroid cultures of MCF‐7R were performed to have a more reliable model for studying drug penetration as compared with monolayer cells. For the first time, we observed the behavior of multidrug‐resistant cells grown in a 3D model after treatment with HNTs‐Ht nanomaterial. Treatment with HNTs‐Ht/Cur/Doxo nanomaterial provoked a partially disaggregation; the volume of tumor spheroid after 3 days of treatments increased compared with the untreated spheroids as a consequence of cell detachment. Doxorubicin instead reduced the size and increase the density of tumor spheroid as a sign of diffused cell damage in a dose‐dependent manner (Figure 5a,b).

The cytotoxic activity of HNTs‐Ht/Cur/Doxo was evaluated in 3D MCF‐7R by RealTime‐Glo MT Cell Viability Assay, after 72 h of treatment (Figure 5c,d). The IC_50_ value (150.0 ± 7.0 µM) resulted 7.5‐fold higher compared with that obtained in 2D cell lines. This value equates to the concentration of curcumin present in the nanomaterial which is congruent with the concentration of doxorubicin of 3 µg/mL, while IC_50_ doxorubicin free in 3D model results to be 5 µg/mL. Even in the 3D model, the nanomaterial confirms the advantage of determining a synergistic effect between curcumin and doxorubicin. This result, observed for the first time in a 3D cell model, highlights how clay mineral‐based nanomaterials are also functional in a model that closely mimics the in vivo cellular model in terms of response to drugs.

## 3 CONCLUSIONS

Our study demonstrates that the use of curcumin, combined with doxorubicin and delivered through a clay mineral‐based nanomaterial, can effectively overcome MDR in breast cancer cells. Curcumin's ability to enhance doxorubicin accumulation in MCF‐7R cells highlights its significant efficacy in combating drug resistance. The clay mineral‐based nanomaterial not only improves the bioavailability of both curcumin and doxorubicin but also offers a targeted approach to delivering these agents specifically to resistant tumor cells while preserving the physiological function of P‐gp in healthy cells. Noteworthy for the first time the multifunctional system we propose has been tested not only on a 2D but also on a 3D multidrug‐resistant model. Also in this case the nanomaterial allows to obtain a synergistic cytotoxic effect obtained with subcytotoxic concentrations. In summary, the combination of curcumin and doxorubicin in a multifunctional delivery system represents a promising strategy to address MDR in breast cancer. This approach not only enhances therapeutic efficacy but also provides a novel means to resensitize drug‐resistant tumor cells, opening new avenues for cancer therapy.

## 4 MATERIALS AND METHODS

All reagents needed were purchased from Sigma‐Aldrich and used without further purification. HNTs‐SH and Ht‐Allyl were synthesized as previously reported.^[^ ^30^ ^]^

Microwave (MW)‐assisted syntheses were carried out with a CEM DISCOVER monomode system (CEM Corporation) in a closed vessel.

Transmission electron microscopy (TEM) was performed by means of a FEI Titan G2 60–300 ultrahigh‐resolution transmission electron microscope (FEI) coupled with analytical electron microscopy (AEM) performed with a SUPER‐X silicon‐drift windowless energy‐dispersive X‐ray spectroscopy (XEDS) detector. AEM spectra were saved in mode scanning transmission electron microscopy (STEM) with a high‐angle annular dark field (HAADF) detector. X‐ray chemical element maps were also collected. The size analysis and ζ‐potential values of the nanomaterials were determined using a Malvern Zetasizer Nano ZS instrument, fitted with a 532 nm laser at a fixed scattering angle of 173°.

UV–*vis* measurements were performed using a Beckmann DU 650 spectrometer.

Fourier transform infrared spectroscopy (FT‐IR) spectra (KBr) were recorded with an Agilent Technologies Cary 630 FT‐IR spectrometer.

TGA were performed on a Mettler Toledo instrument, equipped with a sensor and FRS5 microbalance (precision 0.1 μg) and FP89 software package, using a heating rate of 5°C min^–1^ in the 30–800°C temperature range.

### 4.1 Synthesis of HNTs‐Ht nanomaterial

HNTs‐SH and Ht‐Allyl, in different proportions, were weighed in a MW test tube provided with a cap, and a catalytic amount of azobisisobutyronitrile (AIBN) was added. The mixture was inserted in the MW apparatus at 100°C, under constant stirring, for 1 h. Successively, the solid was filtered off, rinsed several times with MeOH, and dried at 80°C under vacuum.

### 4.2 Loading of curcumin and doxorubicin on HNTs‐Ht nanomaterial

To a dispersion of HNTs‐Ht (100 mg) in water (5 mL), a solution of curcumin in MeOH (1 ·10^−2 ^M, 2 mL) was added. The obtained dispersion was left under stirring for 18 h at room temperature. After this time, the solvent was filtered off and the obtained powder was washed several times with water and left to dry at 60°C. The supernatant solution was analyzed by means UV–*Vis* spectroscopy at the wavelength of 420 nm. The loading percent (LD%) and entrapment efficiency (EE%) were calculated by the following equation:

(1) $$\text{LD}\%=\frac{{\text{Cur}}_{\text{HNTs}-\text{Ht}}}{m_{\text{HNTs}-\text{Ht}}+{\text{Cur}}_{\text{HNTs}-\text{Ht}}}\times 100,\;$$

(2) $$\mathrm{EE}\%=\frac{{\text{Cur}}_{\text{HNTs}-\text{Ht}}}{{\mathrm{Cur}}_{\mathrm{Tot}}}\times 100,$$

where Cur_HNTs‐Ht_ and Cur_Tot_ are the amount of curcumin loaded on the HNTs‐Ht nanomaterial and the total feed curcumin, respectively, and m_HNTs‐Ht_ is the amount of nanomaterial.

The loading of doxorubicin onto the HNTs‐Ht/Cur nanomaterial was carried out by mixing a nanomaterial aqueous dispersion (50 mg in 2.5 mL of H_2_O) with an aqueous doxorubicin solution (100 mL of a 2 mg mL^‐1^ solution). Then, the obtained suspension was left to stir at room temperature for ca. 6 h. After loading, the supernatant solution was centrifuged off and the amount of drug loaded was estimated by UV–*vis* spectroscopy at the wavelength of 500 nm by using the Lambert–Beer law.

### 4.3 Antiproliferative effects evaluation of nanomaterial

The cytotoxic effects of HNTs/Ht/Doxo/Curc nanomaterial were evaluated in MCF‐7R cells after 72 h of treatments by viable cell count with Trypan blue exclusion test. Data were expressed as mean ± standard error (SE) of two different experiments performed in duplicate.

### 4.4 Cell lines and antiproliferative effects evaluation

MCF‐7 cell line was obtained from ATCC (HTB‐22™). The MDR cell line MCF‐7R was derived by treating the wild‐type cells with doxorubicin. The IC_50_ value of doxorubicin in MCF‐7R is approximately 75 times higher than the original IC_50_. MCF‐7R cells lack ERα expression, is estrogen‐insensitive, overexpresses P‐gp and is characterized by the overactivation of NF‐κB. Cell lines were cultured in Dulbecco's Modified Eagle Medium (DMEM) (HyClone Europe Ltd), supplemented with 10% heat‐inactivated fetal calf serum, 2 mM l‐glutamine, 100 units/mL penicillin and 100 μg/mL streptomycin (all reagents were from HyClone Europe). All cell lines were cultured in a humidified atmosphere of 5% CO_2_ at 37°C. The cultures were routinely tested for Mycoplasma infection. Cells with a narrow range of passage numbers were used for all experiments.

### 4.5 P‐gp ATPase activity determination

P‐gp ATPase activity was performed with Pgp‐Glo™ Assay Systems (Promega) following the manufacturer's instructions. Curcumin (test compound, TC) was added to a 96‐well white plate in duplicate and incubated with recombinant human P‐gp membranes. No treatment control (NT) with only Pgp‐GLO assay buffer was used to provide a measure of unregulated ATPase activity, while Na_3_VO_4_ (0.25 mM) was used as the selective inhibitor of P‐gp ATPase activity and provides a measure of P‐gp‐independent ATPase activity. Verapamil (0.5 mM) is a P‐gp substrate that stimulates P‐gp ATPase activity and serves as a positive control for drug stimulation of P‐gp ATPase activity. We also performed an ATP Standards curve as internal control, to verify the correct execution of the assay. MgATP (5 mM) was added to initiate the ATPase activity. After 40 min incubation at 37°C, the reaction was stopped with 50 µL ATPase Detection Reagent and then incubated for 20 min at room temperature. Luminescence was measured using a GLOMAX Multidetection System (Promega). We calculated ΔRLU_basal_ that reflects basal P‐gp ATPase activity as the difference between the average luminescent signals from Na_3_VO_4_‐treated samples (RLU_Na3VO4_) and untreated (NT) samples (RLU_NT_). After, we calculated ΔRLU_TC_ that reflects P‐gp ATPase activity in the presence of a test compound, as the difference between the average luminescent signals from Na_3_VO_4_‐treated samples (RLU_Na3VO4_) and test compound‐treated samples (RLU_TC_). Verapamil is added in the TC wells, and the results are presented as RLU produced by curcumin on stimulated P‐gp.

### 4.6 Determination of doxorubicin accumulation

To evaluate the effects of curcumin on intracellular accumulation of doxorubicin in MCF‐7R cell line, the cells were cultured in 24‐well plates at a density of 1 × 10^5^ cells per well. After 24 h, the cells were treated with curcumin (30 µM) or verapamil (10 µM). After 24 h of incubation doxorubicin (25 µM) has been added for different time: 1 and 2 h. Subsequently, the cells were washed twice with PBS and then resuspended in 200 µL PBS for doxorubicin fluorescence intensity measurement by flow cytometry using a FACSCanto (Becton Dickinson). The results are presented as a percentage of fluorescence intensity relative to control (means ± SE of two experiments). The same analysis was conducted on the parental cell line.

### 4.7 Microscopic analysis

MCF‐7R cells were seeded at 8 × 10^4^ cells in ibidi 35 mm imaging dishes, for live imaging; once a confluence of 70% was reached, were treated with nanomaterial for 6 and 24 h and observed by 3D Cell Explorer microscope, Nanolive imaging.

For immunofluorescence staining, MCF‐7R were seeded in 8 well Nunc lab tek chamber slide system at a concentration of 5 × 10^3^ cells/well and treated at the same time. After 6 and 24 h the cells were fixed with cold methanol for 20 min, washed with PBS 1× twice for 5 min, and stained with DAPI for 10 min, washed and mounted with mounting medium, and observed with Leica DM2500 microscope.

### 4.8 Spheroid formation and cell proliferation assay in 3D conditions

MCF‐7R cells were seeded at 2 × 10^4^ cells/mL with the addition of type l rat tail collagen, 6 µg/mL (Corning, Sigma Aldrich srl) in a white, 96‐well multiwell plates, 100 µL/well (Costar®3903 white clear bottom, Sigma Aldrich srl), as recommended in kit instructions. The plates were centrifuged at 200 *g* for 3 min at room temperature and placed in an incubation chamber with 5% of CO_2_ at 37°C. After 4 days, the spheroids reached optimal size and shape; those with uniform characteristics were used to evaluate the cytotoxicity of the nanomaterial. The medium was replaced with fresh DMEM for control spheroids not treated (control), and with DMEM with HNTs‐Ht/Doxo/Curc at the indicated concentrations for 72 h. The diameter of spheroids was measured using (Zeiss AX10, 5× magnification) at time 0 and after treatment.

Cell viability was performed by RealTime‐Glo™ MT Cell Viability Assay (G9712 Promega) following the manufacturer's recommendations and luminescence (RLU) using a plate‐reading luminometer (GLOMAX Multidetection System, Promega) was detected. HNTs‐Ht/Doxo/Curc cytotoxicity on 3D MCF‐7R was expressed as fold changes in cell viability compared with control and are presented as mean ± SE of two experiments.

### 4.9 Statistical analysis

The results obtained are reported as mean ± standard error (SE). Statistical analysis was performed by analysis of variance (one‐way ANOVA) followed by Tukey's test. STATISTICS ver. 10 (StatSoft Inc. 2011) was used as the analysis software.

## Supporting information

Supporting information.

## Data Availability

The data that support the findings of this study are available from the corresponding author upon reasonable request.
