# Supplementary material for: Curcumin and doxorubicin encapsulated in biocompatible clay‐based nanomaterial: A strategy to overcome multidrug resistance
Source: Arch Pharm (Weinheim). 2024 Dec 26;358(1):e2400702. doi: 10.1002/ardp.202400702 (PMC11671670; doi:10.1002/ardp.202400702)
Supplement: Supplementary file 1 — Supporting information. [file ARDP-358-e2400702-s001.docx]

SUPPORTING INFORMATION

**Curcumin and Doxorubicin Encapsulated in Biocompatible Clay-based Nanomaterial: a Strategy to Overcome Multidrug Resistance**

Paola Poma,^a^ Marina Massaro,^a^ Salvatrice Rigogliuso,^a^ Lucia Condorelli, ^a^ Rita Sánchez-Espejo,^b^ César Viseras,^b,c^ Monica Notarbartolo*^,a^ and Serena Riela*^,d^

^a.^ Dipartimento di Scienze e Tecnologie Biologiche, Chimiche e Farmaceutiche (STEBICEF), Università di Palermo, Viale delle Scienze, Parco d’Orleans II, Ed. 16-17 90128 Palermo, Italy. Email: monica.notarbartolo@unipa.it.

^b.^ Department of Pharmacy and Pharmaceutical Technology, Faculty of Pharmacy, University of Granada, Campus Universitario de Cartuja, 18071 Granada, Spain.

^c.^ Andalusian Institute of Earth Sciences, CSIC-UGR, 18100 Armilla, Granada, Spain.

^d.^ Dipartimento di Scienze Chimiche (DSC), Università di Catania, Viale Andrea Doria 6, 95125, Catania, Italy. Email serena.riela@unict.it

Total number of pages 5

Total number of Figures 3

**Table of content**

1. Flow cytometry analysis on MCF-7 cell line……………………………………………………...S3

2. Thermogravimetric curves of Ht and Ht/Cur nanomaterials……..……………………………….S4

3. FT-IR spectrum of HNTs-Ht/Cur/Doxo nanomaterial……………………………………………S5


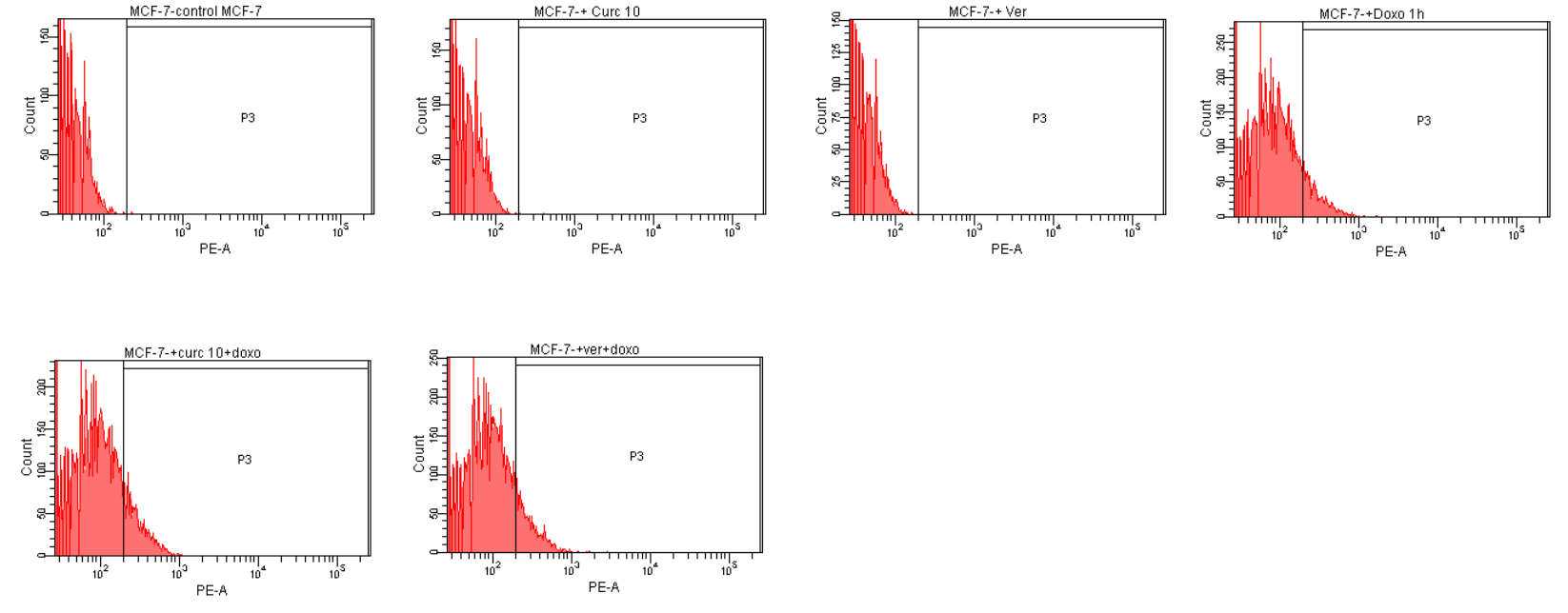


**Figure S1**. Effects of curcumin on intracellular accumulation of doxorubicin in MCF-7 cell line. Representative example of flow cytometry analysis. The cells were treated with curcumin 10 µM or verapamil 10 µM. After 24 h of incubation, doxorubicin 25 µM (13.6 µg/mL) has been added for 1 h.

**Figure S2.** Thermogravimetric curves of Ht and Ht/Cur nanomaterials.

**
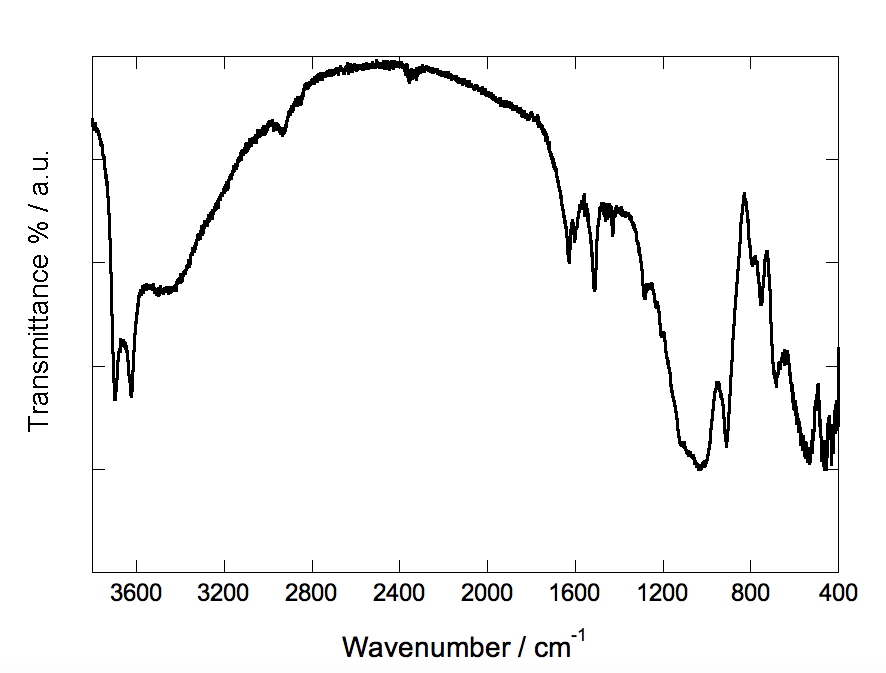
**

**Figure S3.** FT-IR spectrum of HNTs-Ht/Cur/Doxo nanomaterial.
